# Enhancement of Anemia Detection by Correlating Computed Tomography Findings of Abdominal Aorta and Inferior Vena Cava With Laboratory Investigations

**DOI:** 10.7759/cureus.32278

**Published:** 2022-12-07

**Authors:** Mohammad Wazzan, Ahmed Abduljabbar, Amr Ajlan, Khalid Khashoggi, Ayman Eskandar, Turki Alhazmi, Rani Ahmad, Abdulaziz Alotaibi, Ahmed Subki

**Affiliations:** 1 Department of Radiology, Faculty of Medicine, King Abdulaziz University, Jeddah, SAU; 2 Department of Medicine, Umm Al-Qura University, Makkah, SAU; 3 Department of Medical Imaging, Umm Al-Qura University, Makkah, SAU; 4 Department of Pediatric Nephrology, King Fahad Medical City, Riyadh, SAU; 5 Department of Internal Medicine, King Faisal Specialist Hospital and Research Centre, Jeddah, SAU

**Keywords:** abdominopelvic ct scan, hounsfield unit, computed tomography, hemoglobin, anemia

## Abstract

Anemia affects approximately a quarter of the global population, and improved detection may reduce the associated morbidity and mortality. This study investigated correlations between the results of laboratory hematological determinations of hemoglobin levels and attenuation values measured in the lumina of the abdominal aorta and inferior vena cava (IVC) via unenhanced computed tomography (CT) with the aim of expanding diagnostic options for anemia.

The data of 423 patients who underwent abdominal unenhanced CT examinations and laboratory examinations at a tertiary hospital were retrospectively evaluated. CT data were collected using a standard abdominal protocol without contrast. The 151 patients who met the inclusion criteria were categorized by hemoglobin values as follows: <8 (severe anemia), 8-10.9 (moderate anemia), 10.9-12 (mild anemia in females), 10.9-13 (mild anemia in males), and >13 g/dL (non-anemic). The mean CT attenuation values in the aorta and IVC were 37.7 and 36.1 Hounsfield units (HU), respectively. A regression analysis performed to evaluate the correlation and predictability of hemoglobin-based aortic and IVC density yielded a coefficient of determination, R^2^: 0.42 (F ratio: 149.23, p < 0.0001). The highest contribution in the dependent variable (hemoglobin) was reported to IVC density, showing a significant positive correlation between hemoglobin and IVC density.

Our study results demonstrate significant correlations between the densities of the aorta, IVC, and hemoglobin value. Accordingly, radiologists and clinicians can use these readily available values to facilitate diagnosis and patient care.

## Introduction

Computed tomography (CT) is a medical imaging modality in which both software and hardware technologies generate accurate images of the structures within the body from a sequence of X-ray images. CT can efficiently detect various abnormalities in many different internal organs, soft tissues, bones, and blood vessels within a short period. These advantages have led to the widespread implementation of this modality as a diagnostic tool [1-2].

Anemia is a hematological disorder that affects approximately 1.62 billion people worldwide (24.8% of the global population) [3]. Anemia is well known to be associated with increased mortality when accompanied by other diseases, such as pulmonary embolism, as demonstrated by previous studies in which severe anemia was detected using unenhanced CT [4-10]. Currently, hemoglobin (Hb) levels can be easily detected by serum analysis, and more complex diagnostic modalities are not required. However, recent studies have demonstrated the potential use of radiological imaging modalities such as unenhanced CT to diagnose anemia. Such diagnostic methods are based on the measured attenuation values (i.e., densities) of the blood components in Hounsfield units (HU) [5,6,11]. We believe that an increase in the available modalities for detecting anemia, including unenhanced CT, could increase the rate of detection in the early stages and thus prevent further complications while improving patient outcomes [12-13].

Therefore, in this study, we aimed to demonstrate a correlation between serum Hb levels and attenuation values measured within the lumina of the abdominal aorta and inferior vena cava (IVC) during routine unenhanced CT examinations. Our research is novel because although a few studies have demonstrated using HU values on unenhanced CT images to assess anemia, none have attempted to assess the attenuation values of other blood components [4,5]. We believe that unenhanced abdominal CT can be used to determine the anemia status. We suggest using machine learning and artificial intelligence to automatically estimate blood component values in vessels by the HU density to automatically flag patients with anemia. These advancements and results would allow radiologists and clinicians to use readily available measures to facilitate diagnosis and patient care.

## Materials and methods

The King Abdulaziz University Hospital medical record system, Phoenix Health Information System (Richardson, Texas, United States), and diagnostic radiology system, Sectra RIS/PACS "IDS7/dx" (Sectra AB, Linköping, Sweden), were used to identify 423 patients who underwent laboratory and CT examinations. The patient inclusion criteria were as follows: age >15 years, available data from an unenhanced abdominal CT examination, and laboratory evaluations performed within 48 hours before CT. Patients younger than 15 years, those without available unenhanced abdominal CT data, those who underwent laboratory evaluation >48 hours before the CT, and those whose CT images indicated calcification or atherosclerosis of the aorta, beam hardening, or contrast were not included in our analysis. The latter exclusion was set to ensure the selection of slices free of beam hardening, streaks, or motion artifacts.

Data collection

Study data were obtained via unenhanced CT examinations performed using a 64- or 128-slice CT system (Siemens Somatom AS+; Siemens Healthineers, Erlangen, Germany) with a standard abdominal acquisition protocol (100-140 kV, modulated mA). A soft tissue window was used for all CT scans, and the radiologist was oblivious to the laboratory results. Reconstructions were performed using a soft (B30/B31) standard body kernel. The patients did not receive a contrast medium before or during the examination.

**Figure 1:**
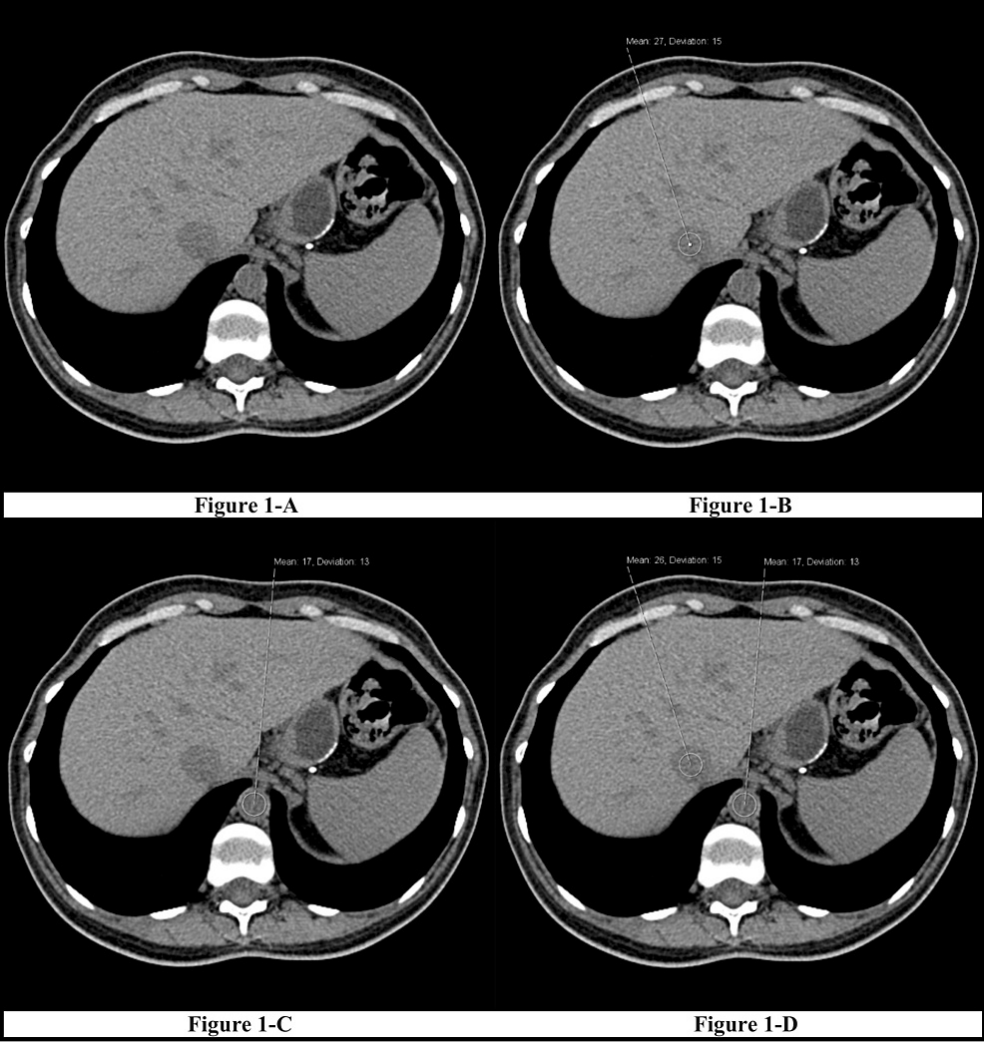
An example of axial CT images with measurements at the IVC and the aorta 1A-1D: Collection of vessel densities for the CT scans; 1A: the general level of the CT scans taken; 1B: Measurement taken from the intrahepatic IVC; 1C: Measurement taken from the abdominal aorta; 1D: Final CT measurements for data collection IVC: inferior vena cava

A student, trained by a consulting radiologist, selected slices that correctly represented the desired anatomical landmarks. Circular regions of interest with two measurements and areas of 1-1.5 cm^2^ were placed on axial slices at the intrahepatic vein level at which the readings were obtained (Figure 1A), intrahepatic IVC (Figure 1B), and abdominal aorta (Figure 1C).

All patients underwent Hb-level analyses within two days before the CT evaluation. Anemia was defined as a Hb level of <12.0 g/dL in women and <13.0 g/dL in men. Mild anemia was defined as a Hb level of 10.9-12 g/dL in women and 10.9-13 g/dL in men. Moderate anemia was diagnosed when the Hb was 8-10.9 g/dL, while severe anemia was diagnosed when the Hb was <8 g/dL [14]. Additional patient variables recorded in the study database were age, gender, aortic density, aortic deviation, IVC density, and IVC deviation.

Statistics

MedCalc Statistical Software, version 17.9.7 (MedCalc, Ostend, Belgium), was used for statistical analyses. The following statistical methods and techniques were used: central tendency measures (means and standard deviations), independent samples t-test, analysis of variance, Pearson’s correlation coefficient, and linear regression. Statistical significance was set at p<0.05.

## Results

The study sample comprised 423 patients with a mean age of 48.4 ± 17.7 years (range, 15-92 years), more than half (56.3%) of whom were males. Most patients (64.3%) were non-anemic. Of the remaining patients, 15.4%, 15.6%, and 4.7% had mild, moderate, and severe anemia, respectively (Table 1). The mean aortic and IVC deviations in all patients were 15.6052 and 15.2766, respectively (Table 2).

**Table 1:** Characteristics of the study participants (n = 423)

Variables	Results % (n)
Sex	
Male	56.3 (238)
Female	43.7 (185)
Anemia	
None	64.3 (272)
Mild	15.4 (65)
Moderate	15.6 (66)
Severe	4.7 (20)
Total	100.0 (423)

**Table 2:** Mean aortic and IVC deviations stratified by sex and anemia severity IVC: inferior vena cava

	Aortic deviation: Mean (SD)	IVC deviation: Mean (SD)
Combined	15.6052 (3.31)	15.2766 (3.43)
Sex		
Male	14.89 (3.175)	15.91 (3.379)
Female	16.16 (3.304)	14.46 (3.328)
Anemia severity		
None	15.63 (3.33)	15.38 (3.52)
Mild	15.34 (3.44)	14.98 (3.32)
Moderate	15.89 (3.12)	15.32 (3.31)
Severe	15.15 (3.31)	14.70 (3.03)

Patients were further stratified according to gender and anemia severity. No significant differences in age were observed according to gender (Table 3). Notably, however, male patients had significantly higher aortic and IVC densities than female patients; (39.3 vs. 37.9, p = 0.006) and (36.9 vs. 35.0, p≤0.001), respectively. Male patients also had a significantly higher mean Hb level than female patients (13.6 g/dL vs. 11.7 g/dL, p≤0.001) (Table 3).

**Table 3:** Mean, standard deviation, and t-test analyses of several patient factors stratified by sex IVC: inferior vena cava

Variable	Range	mean (SD)	Male	Female	Test value	p-value
Age (years)	15–92	48.4 (17.7)	48.2 (17.5)	48.6 (18.1)	−0.265	0.791
Aortic density	20–51	37.7 (5.4)	39.3 (5.5)	37.9 (5.2)	2.745	0.006
IVC density	16–51	36.1 (5.4)	36.9 (5.4)	35.0 (5.3)	3.750	<0.001
Hb level (g/dL)	3.7–17.9	12.8 (2.4)	13.6 (2.2)	11.7 (2.2)	8.390	<0.001

Further stratification of patients by anemia severity revealed a significant correlation between this factor and age. Specifically, patients with severe anemia had the highest mean age (67.7 years), whereas non-anemic patients had the lowest mean age (44.7 years; p≤0.001). Patients with severe anemia also had significantly lower aortic and IVC densities than non-anemic patients (31.2 vs. 40.0, p≤0.001, and 25.7 vs. 38.1, p≤0.001, respectively). Patients with severe anemia also had a significantly lower Hb level than non-anemic patients (7.0 vs. 14.2, p≤0.001) (Table 4).

**Table 4:** Mean, standard deviation, and analysis of variance of patient factors stratified by anemia severity IVC: inferior vena cava

Variable	Range	Mean (SD)	Non-anemic (n = 272)	Mild anemia (n = 65)	Moderate anemia (n = 66)	Severe anemia (n = 20)	Test value	p-value
Age (years)	15–92	48.4 (17.7)	44.7 (15.7)	49.5 (18.0)	56.7 (19.7)	67.7 (15.6)	18.974	<0.001
Aortic density	20–51	37.7 (5.4)	40.0 (4.9)	38.6 (5.3)	35.5 (4.8)	31.2 (4.4)	31.625	<0.001
IVC density	16–51	36.1 (5.4)	38.1 (4.3)	35.3 (4.7)	31.7 (4.3)	25.7 (4.4)	79.207	<0.001
Hb level g/dL	3.7–17.9	12.8 (2.4)	14.2 (1.3)	11.9 (0.6)	9.6 (0.9)	7.0 (1.0)	504.633	<0.001

**Figure 2:**
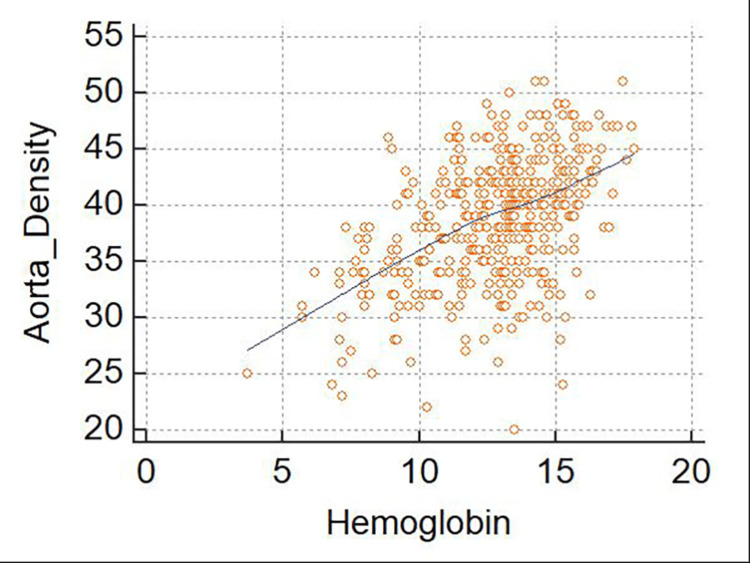
Scatterplot of aortic density as a function of the hemoglobin level This figure shows a significant positive correlation between Hb and aortic density. This correlation is significant (r = 0.467; 95% confidence interval, 0.389–0.538; p≤0.001).

Pearson’s correlation coefficient analyses were performed to test the correlations between Hb levels and aortic and IVC densities. First, the results demonstrated a significant positive correlation between the measured Hb level and aortic density in the general study population (r = 0.467; 95%CI, 0.389-0.538; p≤0.001). When this analysis was stratified by gender, the correlation between the Hb level and aortic density was stronger in female patients (r = 0.585; 95%CI, 0.482-0.673; p≤0.001) than in male patients (r = 0.360; 95%CI, 0.244-0.466; p≤0.001). However, when the analysis was stratified by the anemia severity, the correlation between the Hb level and aortic density remained statistically significant only for non-anemic patients (r = 0.240; 95%CI, 0.124-0.349; p = 0.0001). Although the correlation value was higher among patients with severe anemia, it was not statistically significant (p = 0.083) (Figure 2, Table 5).

**Table 5:** Pearson correlation coefficient (r-value) analysis of the relationship between Hb levels and aortic density

	r-value	95% CI	p-value
Combined patients	0.467	0.389–0.538	<0.0001
Sex			
Male	0.360	0.244–0.466	<0.0001
Female	0.585	0.482–0.673	<0.0001
Anemia severity			
None	0.240	0.1241–0.349	0.0001
Mild	0.095	−0.153–0.331	0.4535
Moderate	0.226	−0.018–0.443	0.0687
Severe	0.397	−0.055–0.714	0.0829

**Figure 3:**
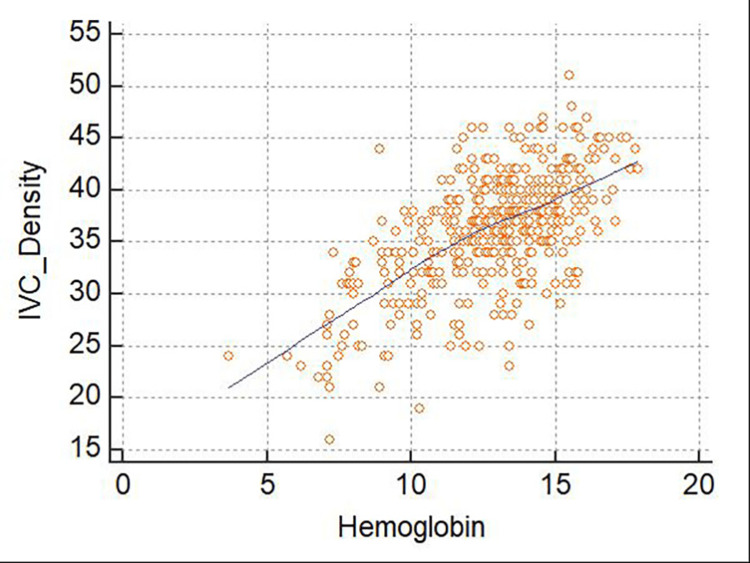
Scatterplot of inferior vena cava (IVC) density as a function of the hemoglobin level This figure depicts a significant positive correlation between Hb and IVC density. This correlation is significant (r = 0.467; 95% confidence interval, 0.389–0.538; p≤0.001).

Similarly, we found a significant positive correlation between the measured Hb level and IVC density in the general study population (r = 0.630; 95%CI, 0.569-0.684; p≤0.001). In a gender-stratified analysis, we observed a stronger correlation between these factors in female patients (r = 0.639; 95%CI, 0.545-0.717; p≤0.001) than in male patients (r = 0.600; 95%CI, 0.512-0.676; p≤0.001). In an analysis stratified by the anemia severity, the results demonstrated that this correlation remained significant only among non-anemic patients (r = 0.316; 95%CI, 0.205-0.419; p<0.001). Again, although the correlation coefficient between Hb and IVC density was higher for patients with severe anemia, the correlation itself was found to be insignificant (p = 0.1297) (Figure 3, Table 6).

**Table 6:** Pearson correlation coefficient (r-value) analysis of the relationship between Hb levels and IVC density

	r-value	95% CI	p-value
Combined patients	0.630	0.569–0.684	<0.0001
Sex			
Male	0.600	0.512–0.676	<0.0001
Female	0.639	0.545–0.717	<0.0001
Anemia severity			
None	0.316	0.205–0.419	<0.0001
Mild	0.109	−0.139–0.344	0.3877
Moderate	0.201	−0.044–0.422	0.1065
Severe	0.351	−0.109–0.687	0.1297

Finally, a linear regression analysis was performed to evaluate the correlation and predictability of hemoglobin-based aortic and IVC densities. The results revealed that the coefficient of determination, R2, was 0.42 (F ratio = 149.23, p≤0.001), and the highest contribution in the dependent variable (Hb) was reported to IVC density. Specifically, the IVC exhibited a greater difference in the HU cutoff values between mild and severe anemia (35.3-25.7 HU) than the difference between these values in the abdominal aorta (38.6-31.2 HU).

## Discussion

The current study aimed to determine whether we could use the blood Hb level to correlate with the densities of the abdominal aorta and IVC lumina on unenhanced routine CT to accurately diagnose anemia. We further aimed to determine any correlations between these parameters with respect to the stratification of cases by the anemia severity levels set forth by the World Health Organization (WHO) [14]. Although previous studies demonstrated that unenhanced CT images of the thorax [4,5,7,8] and head could be used to diagnose anemia [15], only one study by Collins et al. aimed to determine the feasibility of abdominal CT analysis of the aorta and IVC as a means of diagnosing anemia [9]. Although the study found that unenhanced abdominal CT may indicate the presence of anemia [9], the patients were stratified as anemic vs. non-anemic. However, they were not further stratified by the anemia severity or gender.

Consistent with the earlier findings reported by Collins et al. [9], our study showed a significant direct linear correlation between CT attenuation in the abdominal aorta and IVC and the Hb level. However, in contrast to the previous study, we overcame the patient stratification limitation by classifying the patients according to the anemia severity and gender. Accordingly, we observed that patients with severe anemia had significantly lower aortic and IVC densities than non-anemic patients, consistent with previous studies demonstrating a positive correlation between the HU density of blood within vessels and the Hb level [4-6]. Furthermore, we observed a stronger association between the aortic density and Hb levels in women than in men. However, this pattern was statistically significant only among non-anemic patients. This observed difference between male and female patients is consistent with a previous study [10]. This might be attributed to differences in the WHO anemia classification values for men and women [14]. Similarly, the correlation between the IVC density and Hb level was positively significant and stronger in women than in men. Furthermore, a comparison based on anemia severity showed that the gender-related correlation pattern was significant only among non-anemic patients.

Finally, we conducted a linear regression analysis to evaluate the correlation and predictability of hemoglobin-based aortic and IVC densities. The results revealed that the coefficient of determination, R2, was 0.42 (F ratio = 149.23, p≤0.001), and the highest contribution in the dependent variable (Hb) was reported to IVC density. According to this result, the density of the IVC detected anemia more accurately than the abdominal aorta. These results are consistent with those reported previously by Collins [9]; namely, routine abdominal CT examinations can feasibly be used to diagnose anemia.

Regarding the economic aspect of performing abdominal CT for diagnosing anemia, it can be used as an additional informative tool, especially in patients with cancer who are prone to anemia, wherein they will have to undergo CT for follow-up of cancer, thus making CT mandatory and costless by applying artificial intelligence to flag patients with anemia.

Abdominal CT is readily available in hospital radiological systems. In this context, detecting anemia using this modality could help radiologists alter or limit the differential diagnosis.

Our study has some limitations, including those inherent to a retrospective study design, such as the reliance on the availability and preciseness of the medical records and laboratory investigations in the hospital, and the difficulty of controlling confounders and bias due to the lack of randomization or blinding. The results, at best, generate a hypothesis to be then tested prospectively.

Regarding selection bias, our patients may not have been representative of the general population of Saudi Arabia. Although the study sample was normally distributed, there were relatively few cases of severe anemia (Hb <8 g/dL) and fewer cases of anemia (151) than those without anemia (272), which may have altered the CT attenuation cutoff values for determining anemia severity. We further noted that our strict exclusion criteria and, consequently, the number of patients included in the study (423 patients) presented a challenge. Of the 1466 patient CT scans performed in 2016, 1043 were excluded from the sample because the cases met one of the exclusion criteria.

## Conclusions

Current findings suggest that unenhanced abdominal CT can be used to determine the anemia status. Our results further substantiate the significant correlations between abdominal aortic density, IVC density, and Hb levels identified in a previous study. In future research, we may investigate whether the patient's body mass index affects the determination of anemia (e.g., changes in HU values). We should also consider using machine learning and artificial intelligence to roughly and automatically estimate blood component values in vessels by the HU density to automatically flag patients with anemia. These advancements and results would allow radiologists and clinicians to use readily available measures to facilitate diagnosis and patient care.
